# Epidemiological and Clinical Profile of Patients With Non-traumatic Subarachnoid Hemorrhage in a Brazilian Referral Hospital

**DOI:** 10.7759/cureus.100142

**Published:** 2025-12-26

**Authors:** Matheus Ballestero, Thalia S Saraiva, Rodrigo I Pongeluppi, Ricardo S de Oliveira

**Affiliations:** 1 Medicine Department, Federal University of São Carlos, São Carlos, BRA; 2 Division of Neurosurgery, Department of Surgery and Anatomy, Ribeirão Preto Clinics Hospital, Ribeirão Preto Medical School, University of São Paulo, Ribeirão Preto, BRA

**Keywords:** epidemiology, in-hospital mortality, neurologic prognosis, neurosurgery, subarachnoid hemorrhage

## Abstract

Introduction

Non-traumatic subarachnoid hemorrhage (SAH) is a severe neurological condition and remains a public health concern with high morbidity and mortality. Aneurysmal rupture is the primary cause, with modifiable risk factors such as hypertension and smoking playing significant roles. The objectives of this study were to describe the epidemiological, clinical, radiological, and management profile and short-term in-hospital outcomes (mortality and functional status at discharge) of patients with non-traumatic SAH treated at a tertiary neurosurgical referral center in São Carlos, Brazil, between 2016 and 2022, and, secondarily, to examine exploratory, unadjusted associations between baseline clinical severity, in-hospital complications, management strategies (conservative management, coiling, or clipping), and in-hospital outcomes.

Methods

We conducted a retrospective observational cohort study including 125 consecutive patients with non-traumatic SAH. Cases were identified through screening of International Classification of Diseases, Tenth Revision (ICD-10) codes, followed by manual chart review and imaging confirmation. Demographic data, vascular risk factors, clinical presentation, neuroimaging findings, treatment modalities (clinical management, endovascular coiling, or surgical clipping), complications, and in-hospital outcomes (mortality and Modified Rankin Scale (mRS) score at discharge) were extracted from electronic medical records. Descriptive statistics were used to summarize the cohort, and unadjusted comparisons were performed using the Mann-Whitney U test, Kruskal-Wallis test, and chi-square test, with a significance level of p < 0.05.

Results

The mean age of patients was 56 years, and 70% of patients were female. Hypertension (51%), smoking (26%), and alcohol consumption (16%) were common risk factors. Aneurysmal SAH accounted for 65% of cases, predominantly involving the anterior communicating artery. The overall in-hospital mortality rate was 45% (56/125). In unadjusted analyses, in-hospital death was more frequent among patients requiring endotracheal intubation, ventricular shunting, or who developed hydrocephalus, and less frequent among those who received nimodipine. Functional outcome at discharge was poor, with 45% of patients classified as mRS 6 (death) and 15% having moderate-to-severe disability.

Conclusion

In this Brazilian tertiary referral center, non-traumatic SAH predominantly affected middle-aged women, with hypertension and smoking as the main modifiable risk factors. In-hospital mortality was high and was associated with markers of greater clinical severity and complications, such as mechanical ventilation, hydrocephalus, and the need for ventricular shunting. In unadjusted, exploratory analyses, patients undergoing aneurysm coiling or clipping had more favorable crude outcomes than those managed conservatively; however, these differences are likely influenced by baseline severity and treatment selection and should not be interpreted as evidence of treatment efficacy. These findings underscore the need for improved early recognition, control of vascular risk factors, and expanded access to specialized neurosurgical and neurocritical care in resource-limited settings.

## Introduction

Non-traumatic subarachnoid hemorrhage (SAH) is a serious neurological disorder, accounting for 2%-7% of all hemorrhagic strokes worldwide, with approximately 600,000 cases reported annually [1].​ While its global incidence has remained stable, Brazil continues to face high morbidity and mortality rates associated with SAH [2].

The leading cause of non-traumatic SAH is aneurysmal rupture, responsible for 80% of cases. Most aneurysms occur in the anterior circulation of the Circle of Willis, underscoring the role of modifiable risk factors such as hypertension and smoking, along with genetic predispositions [3]. Despite advances in diagnostic imaging and treatment, SAH remains a significant public health challenge in Brazil, imposing not only a heavy psychological and physical burden on patients and their families but also a considerable financial strain on the healthcare system [4].

The primary objective of this study was to describe the epidemiological, clinical, radiological, and management profile and short-term in-hospital outcomes (mortality and functional status at discharge) of patients with non-traumatic SAH treated at a regional tertiary neurosurgical referral center in São Carlos, São Paulo, Brazil, between 2016 and 2022. Our secondary, exploratory objective was to examine unadjusted, hypothesis-generating associations between baseline clinical severity, in-hospital complications, management strategies (conservative management, coiling, or clipping), and in-hospital outcomes, without adjustment for potential confounders. These analyses were descriptive and not intended to test causal hypotheses.

By addressing the scarcity of hospital-based SAH cohorts from Brazilian regional centers and describing management patterns within a resource-limited setting, we aim to provide context-specific data that may help guide targeted interventions to mitigate modifiable risk factors, optimize management strategies, and inform planning of neurosurgical care and rehabilitation services in similar healthcare systems.

## Materials and methods

This study was conducted in accordance with the STROBE (Strengthening the Reporting of Observational Studies in Epidemiology) guidelines. Every aspect of the reporting, including study design, data collection, analysis, interpretation, and presentation of results, complied with the recommendations outlined in the STROBE checklist [5].

Study design and setting

This was a retrospective observational cohort study conducted at a tertiary referral hospital (Santa Casa de São Carlos University Hospital) for neurosurgical procedures in São Carlos, São Paulo, Brazil. The hospital serves as the primary neurosurgical referral center for approximately 500,000 people in the region.

Population and sample

We reviewed the medical records of all patients diagnosed with non-traumatic SAH who were admitted between 2016 and 2022. To maximize sensitivity for potential SAH cases, we first screened all admissions coded with the following International Classification of Diseases, Tenth Revision (ICD-10) codes [6]: I60, I61, I62.9, I66.9, I69.0, and I69.4. In a second step, each of these records was manually reviewed by the study team.

Non-traumatic SAH was confirmed when (1) subarachnoid blood was documented on neuroimaging (non-contrast cranial CT and/or CT/MR angiography or digital subtraction angiography (DSA)), and (2) there was no history of recent head trauma. Patients with traumatic SAH and those with alternative diagnoses (other hemorrhagic or non-hemorrhagic stroke) were excluded. All eligible patients who met the radiological and clinical definitions of non-traumatic SAH during the study period were included; even when some clinical, imaging, or outcome variables were incompletely documented, cases were retained and analyzed on an available-case basis.

Aneurysmal SAH was defined by the presence of a ruptured intracranial aneurysm on angiographic studies (DSA or CT/MR angiography), as interpreted by the treating neuroradiologist or neurosurgeon. Non-aneurysmal SAH was defined as SAH without an identifiable aneurysm or arteriovenous malformation on complete vascular imaging, including perimesencephalic SAH. In selected cases, lumbar puncture and magnetic resonance imaging were used to confirm SAH when CT scans were inconclusive. When non-traumatic SAH was confirmed on CT, but vascular imaging (CT/MR angiography and/or DSA) was incomplete, not performed, or inconclusive, the etiology was coded as "undetermined etiology", precluding a definitive classification as aneurysmal or non-aneurysmal SAH, and these patients were retained in the main analyses.

Data collection

Data were collected from electronic medical records using a standardized electronic data collection form. The following variables were extracted: age, sex, comorbidities (including systemic arterial hypertension, diabetes mellitus, and other vascular risk factors), lifestyle habits (smoking and alcohol use), clinical presentation (symptoms and neurological status), pupil findings, Glasgow Coma Scale (GCS), Hunt-Hess (HH), World Federation of Neurosurgical Societies (WFNS), and Fisher scale scores, neuroimaging findings (CT and angiographic studies), SAH etiology (aneurysmal vs. non-aneurysmal vs. undetermined), treatment modality (clinical management, endovascular coiling, or surgical clipping), complications (such as vasospasm and hydrocephalus), and outcomes.

The primary outcomes were in-hospital mortality during the index admission and functional status at hospital discharge, assessed using the Modified Rankin Scale (mRS). No post-discharge follow-up data were available.

Two trained investigators independently extracted data from the medical records. Discrepancies were resolved by consensus with a third senior investigator (neurosurgeon). Because this was a retrospective chart review, data abstractors had access to the full medical record and were not blinded to in-hospital outcomes, and no formal inter-rater reliability statistics were calculated. To minimize transcription errors, a random sample of records was re-checked for accuracy. No imputation was performed for missing data; analyses were carried out on an available-case basis for each variable, and denominators therefore vary across some analyses.

Ethical considerations

The study received approval from the Research Ethics Committee of the Federal University of São Carlos (approval number 5.774.929). Patient confidentiality was upheld throughout the study, and no identifying information was included in the data.

Treatment modalities

Therapeutic strategies included pharmacological management with nimodipine for the prophylaxis of vasospasm and neurointerventional procedures such as endovascular embolization or surgical clipping of ruptured aneurysms. Ventricular drainage was performed in cases of hydrocephalus when clinically indicated. For the purposes of this study, "clinical (conservative) management" was defined as the absence of any aneurysm-targeted procedure (no coiling or clipping) during the index admission and included (1) patients with very poor baseline neurological status or extensive hemorrhage who were judged unsuitable for intervention; (2) patients with angiographically negative or undetermined etiology SAH and (3) patients for whom aneurysm repair was not available within an acceptable time frame due to local resource constraints. Endovascular coiling and surgical clipping groups comprised patients who underwent the respective aneurysm repair procedures during the index hospitalization. Because the exact timing from ictus to aneurysm repair could not be reliably determined for all patients, we did not categorize procedures as "early" or "delayed".

Statistical analysis

Data were compiled using Microsoft Excel (Microsoft Corporation, Redmond, USA), and analyses were performed with IBM SPSS Statistics (IBM Corp., Armonk, USA) and JASP software (University of Amsterdam). Categorical variables are presented as absolute and relative frequencies, and continuous variables as means with standard deviations or medians with interquartile ranges, as appropriate.

The distribution of continuous variables was evaluated using the Kolmogorov-Smirnov (K-S) test (given the sample size > 50) and visual inspection of histograms and Q-Q plots. Because most variables did not follow a normal distribution, we primarily used non-parametric tests (Mann-Whitney U and Kruskal-Wallis tests) for comparisons of continuous or ordinal variables. Categorical variables were compared using the chi-square test.

All analyses were exploratory and unadjusted. We did not construct multivariable regression models due to the limited sample size, number of events, and proportion of missing data, which would likely produce unstable models. When the Kruskal-Wallis test indicated a statistically significant global difference across more than two groups, we conducted exploratory pairwise post hoc comparisons using Mann-Whitney U tests with Bonferroni adjustment; the corresponding post hoc p-values are also reported. No further formal correction for multiple comparisons was applied; therefore, p-values should be interpreted with caution, and statistically significant findings should be considered hypothesis-generating rather than confirmatory. Statistical significance was set at p < 0.05.

## Results

**Figure 1:**
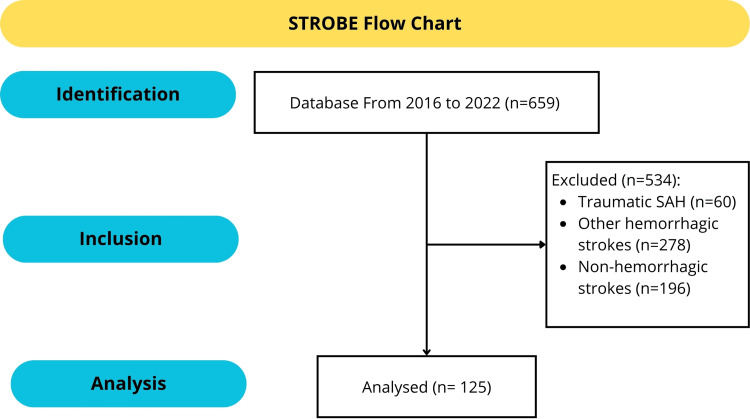
STROBE flowchart of patient selection and study design STROBE: Strengthening the Reporting of Observational Studies in Epidemiology; SAH: subarachnoid hemorrhage

Initially, a total of 659 medical records were selected for evaluation using ICD-10. Following a review, 534 cases (81%) were excluded. The reasons for exclusion were as follows: a history of traumatic SAH (60 cases; 9.1%), classification as other hemorrhagic strokes (278 cases; 42.2%), and diagnoses of non-hemorrhagic strokes (196 cases; 29.7%). Ultimately, 125 eligible individuals (19%) were included in the final analysis for this study (Figure 1).

In this study, the mean age of individuals affected by non-traumatic SAH was 56 years. A significant proportion of patients were female (n=88; 70%), with a male-to-female ratio of 1:2.38. Hypertension was present in 64 (51%) of cases, while 32 (25%) of patients were smokers and 20 (16%) consumed alcohol. Aneurysmal SAH accounted for 81 (65%) cases, with most aneurysms occurring in the anterior communicating artery. Among patients with SAH, 97 (78%) reported experiencing an acute headache episode, while motor deficits, neck stiffness, and seizures were also common symptoms.

The clinical severity of SAH varied among patients. According to the GCS, 72 (58%) exhibited mild neurological deficits (score 13-15), 16 (13%) had moderate deficits (score 9-12), and 37 (nearly 30%) presented with severe deficits (score 3-8). The WFNS scale showed a bimodal distribution, with 44 (35%) classified as grade 1, 23 (18%) as grade 2, and 32 (26%) as grade 5, indicating severely impaired consciousness. Based on the HH scale, 38 (30%) were classified as grade 5, followed by 27 as grade 2 (22%), and 26 as grade 1 (21%). The Fisher scale assessment showed that 57 (46%) patients were Fisher grade 4, while 23 (18%) were Fisher grade 3.

Regarding diagnostic methods, 87 (70%) patients underwent both non-contrast cranial CT and angiography. The underlying cause of SAH could not be determined in approximately one-third of the cases, despite angiographic evaluation. Aneurysm rupture was the leading cause, with some cases involving multiple aneurysms.

In terms of treatment, 108 (86%) of patients received nimodipine, while surgical management included endovascular embolization (n=42; 33%) and aneurysm clipping (n=23; 18%). Nearly half (n=60, 48%) were managed conservatively. Additionally, 32 (26%) of patients required ventricular shunting for hydrocephalus.

Complications were common, with 64 (51%) patients experiencing SAH-related complications, primarily vasospasm (n=34; 27%). The overall mortality rate was 45% (n=56), with the average duration from initial care to death of approximately 15 days. The mRS assessment revealed that 39 (31%) of patients had no significant disability (mRS score 0), 19 (15%) had moderate disability (mRS score 4), and 56 (45%) succumbed to the condition (mRS score 6). Neurological sequelae included hemiparesis (n=19; 15%), diplopia (n=5; 4%), and speech difficulties (n=3; 2%).

The results are summarized in Tables 1-5. Table 1 presents the clinical characteristics of the included patients. Table 2 summarizes diagnostic methods used, patient management, and study outcomes. Table 3 reports bivariate comparisons between mRS classification and other variables using the Mann-Whitney U test. Table 4 presents differences in mRS classification across groups using the Kruskal-Wallis test (non-parametric ANOVA). Table 5 presents chi-square analyses comparing death rates across categorical variables.

**Table 1:** Clinical characteristics of patients included in the study WFNS: World Federation of Neurosurgical Societies The data has been represented as frequency (n) and percentage (%).

Clinical feature	Frequency (n)	Percentage (%)
Presence of comorbidities	84	67.2%
Diabetes mellitus	16	12.8%
Systemic arterial hypertension	64	51.2%
Smoking	32	25.6%
Alcoholism	20	16.0%
Family history of aneurysm	3	2.4%
Patients on mechanical ventilation	43	34.4%
Headache	97	77.6%
Motor deficit	19	15.2%
Neck stiffness	28	22.4%
Seizures	26	20.8%
Hypertensive crisis	24	19.2%
Syncope	22	17.6%
Vomiting	11	8.8%
Decreased level of consciousness	8	6.4%
Pupil alteration		
Isocoric and reactive to light	88	70.4%
Anisocoric	15	12.0%
Miotic	11	8.8%
Medium fixed	6	4.8%
Mydriatic	5	4.0%
WFNS scale grade		
I	44	35.2%
II	23	18.4%
III	5	4.0%
IV	21	16.8%
V	32	25.6%
Hunt-Hess scale grade		
I	26	20.8%
II	27	21.6%
III	23	18.4%
IV	11	8.8%
V	38	30.4%
Fisher scale grade		
1	5	4.0%
2	14	11.2%
3	23	18.4%
4	57	45.6%

**Table 2:** Diagnostic methods, patient management, and outcomes CT: computed tomography; CSF: cerebrospinal fluid; SAH: subarachnoid hemorrhage The data has been represented as frequency (n) and percentage (%).

Diagnosis, management, and outcome	Frequency (n)	Percentage
Diagnostic method		
CT	35	28.0%
CT + angiography	87	69.6%
CSF analysis	1	0.8%
MRI	2	1.6%
Etiology of SAH		
Aneurysm	81	64.8%
Arteriovenous malformation	2	1.6%
No information	42	33.6%
Topography of aneurysms		
Anterior communicating artery	33	26.4%
Internal carotid artery	15	12.0%
Middle cerebral artery	14	11.2%
Posterior communicating artery	6	4.8%
Pericallosal artery	6	4.8%
Multiple aneurysms	5	4.0%
Basilar artery	1	0.8%
Ophthalmic artery	1	0.8%
No topography	44	35.2%
Management method		
Nimodipine	108	86.4%
Magnesium sulfate	3	2.4%
Corticosteroids	33	26.4%
Clinical management	60	48.0%
Aneurysm embolization	42	33.6%
Aneurysm clipping	23	18.4%
Ventricular shunt	32	25.6%
Complication		
General complications	64	51.2%
Vasospasm	34	27.2%
Hydrocephalus	29	23.2%
Infection	6	4.8%
Modified Rankin Classification score		
0	39	31.2%
1	3	2.4%
2	2	1.6%
3	1	0.8%
4	19	15.2%
5	5	4.0%
6	56	44.8%

**Table 3:** Differences between Modified Rankin Scale classification as the dependent variable and other variables according to the Mann-Whitney U test SAH: subarachnoid hemorrhage W represents the Mann-Whitney U test statistic. Data are presented as Modified Rankin Scale, SD, n, W, and p-value; results were considered statistically significant at p < 0.05 (calculated using the Mann–Whitney U test). *p < 0.05

	Modified Rankin Scale				
	Mean	SD	n	W	p
Gender					
Women	3.5	2.6	88	1544	0.627
Men	3.8	2.6	37		
High blood pressure					
Yes	3.4	2.6	64	2097	0.447
No	3.7	2.7	61		
Diabetes					
Yes	3.3	2.8	16	912	0.755
No	3.6	2.6	109		
Smoking					
Yes	3.5	2.8	32	1506	0.918
No	3.6	2.6	93		
Alcoholism					
Yes	4.1	2.7	20	877.0	0.215
No	3.5	2.6	105		
Family history of SAH					
Yes	4.3	2.8	3	136	0.423
No	3.6	2.6	122		
Endotracheal intubation					
Yes	5.3	1.7	43	693	< .001
No	2.7	2.6	82		
Nimodipine					
Yes	3.5	2.6	108	1117	0.127
No	4.2	4.8	17		
Magnesium sulfate					
Yes	2.0	3.5	3	234.5	0.379
No	3.6	2.6	122		
Corticosteroid					
Yes	3.9	2.5	33	1436	0.624
No	3.5	2.7	92		
Ventricular shunt					
Yes	5.3	1.4	32	819.5	< .001
No	3.0	2.7	93		
Vasospasm					
Yes	4.8	1.7	34	1142	0.016*
No	3.1	2.8	91		
Hydrocephalus					
Yes	5.3	1.4	29	766	< .001
No	3.0	2.7	96		
Motor deficit					
Yes	3.8	2.5	19	455.0	< .001
No	2.5	2.6	68		
Headache					
Yes	3.3	2.7	97	110.5	0.460
No	2.7	2.3	3		
Neck stiffness					
Yes	3.8	2.5	28	609.0	0.022*
No	2.4	2.6	61		
Yes	3.8	2.5	28		
Comorbidities					
Yes	3.4	2.6	84	1903	0.321
No	3.9	2.6	41		

**Table 4:** Differences in Modified Rankin Scale classification as the dependent variable and other variables according to the Kruskal-Wallis (non-parametric ANOVA) test Clin: clinical treatment; Clip: surgical clipping; Coil (endovascular coiling); F: ANOVA F test; Fixed: fixed pupils; GCS: Glasgow Coma Scale; Iso: isocoric pupils; Midr: mydriatic pupils Data are presented as mean Modified Rankin Scale scores, SDs, n, %, F, and p-values; results were considered statistically significant at p < 0.05 (calculated using the Kruskal–Wallis test). Post hoc p-values correspond to exploratory pairwise comparisons between groups (Mann–Whitney U tests with Bonferroni adjustment). *p < 0.05

	Modified Rankin Scale									
Treatment		Mean	SD	n	Percentage	F	p	Post-test		p
Clinical		4.7	2.5	60	48.0%	9.260	< .001		Clin x Coil	< .001
Coiling		2.6	2.4	42	33.6%				Clin x Clip	0.001
Clipping		2.8	2.6	23	18.4%				Coil x Clip	0.333
Pupils										
Isochoric		2.8	2.6	88	70.4%	8.064	< .001		Iso x Aniso	< .001
Anisochoric		5.4	1.6	15	12.0%				Iso x Midr	0.002
Mydriasis		6.0	0.0	5	4.0%				Iso x Mios	0.007
Miosis		4.8	2.0	11	8.8%				Iso x Fixed	< .001
Fixed		6.0	0.0	6	4.8%					
Aneurysm topography										
Anterior communicating artery		2.9	2.6	33	26.4%	1.285	0.270			
Posterior communicating artery		3.5	2.8	6	4.8%					
Middle cerebral artery		4.3	2.5	14	11.2%					
Internal carotid artery		2.3	2.6	15	12.0%					
Pericallosal artery		4.2	2.2	6	4.8%					
Ophthalmic artery		0.0	0.0	1	0.8%					
Multiple arteries		3.4	1.9	5	4.0%					
Basilar artery		0.0	0.0	1	0.8%					
GCS score										
15-13		2.4	2.6	72	57.6%	24.16	< .001		13 to 15 x 9 to 12	0.027*
9-12		4.0	2.6	16	12.8%				13 to 15 x 3 to 8	< .001
3-8		5.6	1.2	37	29.6%				9 to 12 x 3 to 8	0.053*
Hunt and Hess scale grade										
I		1.2	2.2	26	20.8%	16.92	< .001		I x III	< .001
II		2.4	2.5	27	21.6%				I x IV	0.009*
III		4.4	2.2	23	18.4%				I x V	< .001
IV		4.1	2.2	11	8.8%				II x III	0.003*
V		5.3	1.8	38	30.4%				II x V	< .001
									III x V	0.037*
									IV x V	0.016*
Fisher scale grade										
1		2.8	2.6	5	4.0%	7.767	< .001		Grade 1 x Grade 4	0.018
2		2.3	2.1	14	11.2%				Grade 2 x Grade 4	< .001
3		3.0	2.8	23	18.4%				Grade 3 x Grade 4	< .001
4		4.9	2.0	57	45.6%					

**Table 5:** Chi-square test analysis of death rates for categorical variables Data are presented as absolute values and percentages, with χ² and p-values; results were considered statistically significant at p < 0.05 (calculated using the chi-square test). *p < 0.05

	Death			
	No	Yes	Chi-square test	
Gender				
Feminine	49 (55.7%)	39 (44.3%)	χ² = 0.028	p = 0.867
Masculine	20 (54.1%)	17 (45.9%)		
Motor deficit	10 (52.6%)	9 (47.4%)	χ² = 4.762	p = 0.029*
Neck stiffness	16 (57.1%)	12 (42.9%)	χ² = 3.677	p = 0.055
Diabetes	9 (56.3%)	7 (43.8%)	χ² = 0.008	p = 0.928
Hypertension	38 (59.4%)	26 (40.6%)	χ² = 0.924	p = 0.336
Smoking	17 (53.1%)	15 (46.9%)	χ² = 0.075	p = 0.784
Alcoholism	8 (40.0%)	12 (60.0%)	χ² = 2.224	p = 0.136
Family background	1 (33.3%)	2 (44.8%)	χ² = 0.594	p = 0.441
Endotracheal intubation	7 (16.3%)	36 (83.7%)	χ² = 40.15	p < .001*
Nimodipine	64 (59.3%)	44 (40.7%)	χ² = 5.291	p = 0.021*
Magnesium sulfate	2 (66.7%)	1 (33.3%)	χ² = 0.163	p = 0.686
Corticosteroid	18 (54.5%)	15 (45.5%)	χ² = 0.008	p = 0.930
Ventricular shunt	10 (31.3%)	22 (68.8%)	χ² = 9.976	p = 0.002
Vasospasm	17 (50.0%)	17 (50.0%)	χ² = 0.511	p = 0.475
Hydrocephalus	8 (27.6%)	21 (72.4%)	χ² = 11.64	p < .001*
Treatment				
Clinical	17 (28.3%)	43 (71.7%)	χ² = 34.06	p < .001*
Coiling	34 (82.9%)	7 (17.1%)		
Clipping	18 (75.0%)	6 (25.0%)		

## Discussion

The epidemiological profile observed in this study is consistent with prior research, highlighting a predominance of middle-aged female patients [7,8]. The higher incidence of SAH in postmenopausal women has been attributed to hormonal factors, particularly estrogen depletion, which may contribute to vascular endothelial dysfunction and aneurysm formation [9,10].

Hypertension (51%) and smoking (25%) emerged as the most relevant modifiable risk factors. The strong association between hypertension and SAH has been well documented, with chronic arterial pressure contributing to aneurysm formation and rupture due to increased hemodynamic stress [11,12]. Similarly, tobacco use is known to promote endothelial dysfunction and inflammation, facilitating aneurysm instability [13]. The prevalence of alcohol consumption (16%) aligns with previous studies linking excessive alcohol intake to an elevated risk of SAH [14].

The most common symptom reported was acute headache (78%), which remains the cardinal clinical feature of SAH [15,16]. The relatively low rate of reduced consciousness (6%) observed in this study contrasts with earlier findings, where altered mental status was reported in up to 53% of patients [15,16]. This discrepancy may be due to variations in disease severity at presentation, prompt medical intervention, or differences in referral patterns.

Neuroimaging findings corroborate current best practices, with 70% of patients undergoing both non-contrast cranial CT and angiographic evaluation. While CT remains the primary diagnostic tool for SAH, with a sensitivity exceeding 95% when performed within six hours [17], the relatively high proportion (33%) of patients with undetermined etiology likely reflects the limited feasibility or availability of complete vascular imaging in some cases, particularly among very severe or clinically unstable patients, and indicates that a substantial subset of non-traumatic SAH cases in our setting could not be definitively classified as aneurysmal or non-aneurysmal and may include perimesencephalic hemorrhages [18].

Aneurysmal rupture was the most frequent etiology (65%), predominantly affecting the anterior circulation. This distribution aligns with established anatomical patterns, as aneurysms in the anterior communicating artery are among the most prone to rupture [19]. The lower prevalence of multiple aneurysms (4%) compared to other reports (up to 30%) [20] may reflect population-specific factors or differences in imaging sensitivity.

The choice between surgical clipping and endovascular embolization remains a subject of debate. While this study found no significant difference in mortality between the two approaches, the literature suggests that embolization is associated with lower procedural morbidity, whereas clipping may offer superior long-term durability [21,22]. Notably, the high rate of conservative management (48%) likely reflects cases where patients were deemed unsuitable for surgical intervention due to poor clinical status or extensive hemorrhage. Consequently, the more favorable crude, unadjusted outcomes observed in patients treated with coiling or clipping than in those managed conservatively likely reflect the selection of patients with more favorable baseline conditions; these exploratory associations cannot be interpreted as evidence of superiority of any specific treatment modality.

Given the limited sample size and the unadjusted nature of our analyses, the study was not powered to detect subtle differences between clipping and coiling, and any apparent differences should be regarded as exploratory.

Complications remain a major concern in SAH management. Vasospasm was the most frequent late complication (27%). This rate is somewhat lower than the 40%-70% reported in many series using routine angiographic or transcranial Doppler surveillance [23], and may reflect differences in diagnostic criteria, monitoring intensity, or case mix in our real-world setting. In prior studies, vasospasm has been identified as a leading cause of delayed cerebral ischemia, with factors associated with higher risk including younger age, female sex, smoking, and high Fisher scale scores [23].

Hydrocephalus was identified in 26% of patients, necessitating ventricular drainage in many cases. In previous reports, hydrocephalus has been associated with a substantially increased mortality risk (up to 60-fold) [24], which emphasizes the need for vigilant monitoring and early intervention. The pathophysiology of post-SAH hydrocephalus remains incompletely understood but is thought to involve inflammatory responses, impaired CSF absorption, and blood product accumulation within the ventricular system [24].

Pupillary abnormalities were significantly associated with poor outcomes, reinforcing their prognostic value. Prior studies have demonstrated that anisocoria or fixed pupils at admission strongly correlate with mortality and long-term disability [25].

Functional outcomes, as assessed by the Modified Rankin Scale, revealed substantial morbidity and mortality, with 45% of patients classified as mRS 6 (death) and 15% experiencing moderate-to-severe disability. These findings align with previous studies, where mortality rates range from 17% to 50% and a significant proportion of survivors experience long-term neurological impairment [26].

Our in-hospital mortality of 45% lies toward the upper end of the range reported in contemporary SAH studies. This high mortality is probably related to several characteristics of our cohort: many patients presented with poor clinical grades at admission, nearly half were managed conservatively (48%), and in some cases, local resource constraints may have delayed or limited access to early aneurysm repair and advanced neurocritical care. These contextual factors should be taken into account when comparing our outcomes with those from high-income settings. Similarly, the lower crude mortality observed among patients who received nimodipine in our cohort should be interpreted as an unadjusted exploratory finding, susceptible to confounding by indication, rather than as evidence of a protective causal effect of the drug.

Although our study did not assess long-term outcomes, previous research has shown that the consequences of SAH extend beyond physical disability, with cognitive deficits, depression, and reduced quality of life affecting up to one-third of survivors [27,28]. These external findings highlight the need for comprehensive rehabilitation programs, including cognitive and psychological support, to enhance functional recovery and long-term well-being.

Future research should focus on refining treatment algorithms, optimizing neurosurgical decision-making, and evaluating emerging neuroprotective strategies. Additionally, advances in imaging modalities and biomarker research may enhance early detection and risk stratification of aneurysmal SAH [29].

Strengths and limitations

This study included all consecutive non-traumatic SAH admissions over a seven-year period in a regional tertiary neurosurgical center in a middle-income country, with a systematic collection of detailed epidemiological, clinical, radiological, and management data using standardized scales (GCS, Hunt-Hess, WFNS, Fisher, and mRS), focusing on real-world care in a resource-limited setting. This enhances the external validity and contextual relevance of our descriptive findings and facilitates comparison with other SAH cohorts and future meta-analyses.

This study also has several limitations. First, its retrospective, single-center design, based on a review of existing medical records, introduces the possibility of information and documentation bias. The accuracy of some clinical parameters and outcomes may have been affected by incomplete or inconsistent charting. Second, case identification relied on an initial ICD-10-based screening step, followed by manual confirmation of non-traumatic SAH. Although this two-step process was designed to maximize sensitivity, a large proportion of initially screened records were excluded, and some degree of misclassification may remain despite detailed chart review. Third, missing data were frequent for some variables, including SAH etiology, which was undetermined in approximately one-third of cases. In many of these patients, complete vascular imaging was not feasible or was not performed, often because they were clinically too unstable or their condition deteriorated rapidly, or due to local resource constraints, so a definitive aneurysmal versus non-aneurysmal cause could not be established. We did not perform data imputation, and analyses were conducted on an available-case basis; as a result, denominators vary, some estimates may be biased, and the true distribution of etiologies in our population should be interpreted with caution. Fourth, comparisons between clinical management and neurosurgical interventions (coiling or clipping) are at high risk of confounding by indication. Patients with better baseline neurological status are more likely to be selected for surgical or endovascular treatment, whereas those with catastrophic presentations are often managed conservatively. Because we did not perform multivariable regression or adjust for baseline differences, the observed associations between management strategies and outcomes should not be interpreted as causal. Fifth, multiple statistical tests, including exploratory post hoc pairwise comparisons, were performed with only limited adjustment for multiple comparisons (simple Bonferroni correction for pairwise tests), increasing the overall risk of type I error. The findings should therefore be interpreted primarily as descriptive and hypothesis-generating, rather than as definitive evidence regarding prognostic factors or comparative effectiveness.

Finally, we only assessed in-hospital outcomes; no long-term functional, cognitive, or quality-of-life data were available. Our findings thus reflect short-term hospital course and cannot be extrapolated to long-term prognosis.

## Conclusions

Understanding the epidemiological and clinical profile of patients with SAH in developing countries is essential for guiding public health policies and healthcare investments. In this retrospective cohort from a Brazilian tertiary referral center, non-traumatic SAH predominantly affected middle-aged women, with hypertension and smoking identified as important modifiable risk factors. In-hospital mortality was high, particularly among patients with markers of greater clinical severity and complications such as hydrocephalus, need for ventricular drainage, and mechanical ventilation.

In unadjusted, exploratory analyses, patients undergoing aneurysm coiling or clipping had more favorable crude outcomes than those managed conservatively; however, these differences are likely influenced by baseline clinical status and treatment selection and should not be interpreted as evidence of treatment efficacy. Our findings underscore the need for improved early recognition, aggressive control of vascular risk factors, and expanded access to specialized neurosurgical and neurocritical care in resource-limited settings. Future prospective studies with larger samples, multivariable adjustment, and long-term follow-up are needed to refine risk stratification and evaluate interventions to reduce the burden of SAH.
